# Supplementary material for: Aesthetic dental treatment, orofacial appearance, and life satisfaction of Finnish and Brazilian adults
Source: PLoS One. 2023 Jun 29;18(6):e0287235. doi: 10.1371/journal.pone.0287235 (PMC10310051; doi:10.1371/journal.pone.0287235)
Supplement: S1 Table — (DOCX) [file pone.0287235.s001.docx]

**S1 Table. Psychometric indicators related to the fit of the factor models of Orofacial Esthetic Scale (OES), Psychosocial Impact of Dental Aesthetics Questionnaire (PIDAQ), and Satisfaction with Life Scale (SWLS) to the samples.**

|  |  |  | **CFA*** | | | | |  | **Measurement Invariance^§^** | | | | | |
| --- | --- | --- | --- | --- | --- | --- | --- | --- | --- | --- | --- | --- | --- | --- |
|  |  |  |  |  |  |  |  |  | **Sex** | | **Monthly Income** | | **Age** | |
| **Scale** | **Country** | **n** | **λ** | **CFI** | **TLI** | **RMSEA** | **SRMR** | **α** | ΔCFI_M1-M0_ | ΔCFI_M2-M1_ | ΔCFI_M1-M0_ | ΔCFI_M2-M1_ | ΔCFI_M1-M0_ | ΔCFI_M2-M1_ |
| **OES** | Finland^¶^ | 3614 | 0.53-0.89 | 0.95 | 0.92 | 0.13 | 0.04 | 0.89^#^ | -0.002 | -0.006 | -0.002 | -0.005 | -0.002 | -0.009 |
|  | Brazil^¶^ | 3979 | 0.58-0.88 | 0.96 | 0.94 | 0.11 | 0.04 | 0.89^#^ | 0.000 | -0.006 | -0.001 | -0.002 | -0.002 | -0.008 |
| **PIDAQ** | Finland^‡^ | 3614 | 0.64-0.95 | 0.97 | 0.96 | 0.09 | 0.05 | 0.88-0.94^†^ | 0.009 | -0.010 | 0.011 | -0.007 | 0.010 | -0.007 |
|  | Brazil^##^ | 3979 | 0.59-0.95 | 0.96 | 0.96 | 0.08 | 0.05 | 0.87-0.94^†^ | 0.014 | -0.014 | 0.016 | -0.012 | 0.017 | -0.015 |
| **SWLS** | Finland | 3614 | 0.67-0.92 | 0.99 | 0.99 | 0.15 | 0.02 | 0.92^†^ | 0.002 | 0.000 | 0.003 | -0.003 | 0.002 | -0.001 |
|  | Brazil | 3979 | 0.70-0.92 | 0.99 | 0.99 | 0.08 | 0.02 | 0.91^†^ | 0.001 | 0.000 | 0.000 | -0.010 | -0.001 | -0.001 |

*CFA: confirmatory factor analysis, λ: factor loading, CFI: comparative fit index, TLI: Tucker-Lewis index, RMSEA: root mean square error of approximation, SRMR: standardized root mean square residual. #α: Cronbach’s alpha coefficient. †α: ordinal alpha coefficient. §M0: configural model, M1: metric model, M2: scalar model, ΔCFI_M1-M0_: metric invariance, ΔCFI_M2-M1_: scalar invariance. ¶refined model adding a correlation between errors of items 1 and 2. ‡refined model excluding items 9, 13, 14, and 15. ##refined model excluding item 6.
