# Supplementary material for: Aesthetic dental treatment, orofacial appearance, and life satisfaction of Finnish and Brazilian adults
Source: PLoS One. 2023 Jun 29;18(6):e0287235. doi: 10.1371/journal.pone.0287235 (PMC10310051; doi:10.1371/journal.pone.0287235)
Supplement: S2 Table — (DOCX) [file pone.0287235.s002.docx]

**S2 Table. Descriptive statistics of the scores of Orofacial Esthetic Scale (OES) and Psychosocial Impact of Dental Aesthetic Questionnaire (PIDAQ) dimensions and measures of sample adequacy (MSA) for principal component analysis (Finnish sample: n=3,614; Brazilian sample: n=3,979).**

|  | **Sample**: Finnish/Brazilian | | | | | | | |
| --- | --- | --- | --- | --- | --- | --- | --- | --- |
| **Dimension** | Mean | Median | Standard deviation | Minimum | Maximum | Skewness | Kurtosis | MSA |
| **OES** |  |  |  |  |  |  |  |  |
| Satisfaction with Orofacial Appearance | 7.01/7.13 | 7.29/7.43 | 1.58/1.74 | 0/0 | 10/10 | -0.81/-0.88 | 0.58/0.78 | 0.83/0.87 |
| **PIDAQ** |  |  |  |  |  |  |  |  |
| Dental self-confidence | 1.95/2.01 | 2.00/2.00 | 1.02/1.06 | 0/0 | 4/4 | -0.09/-0.09 | -0.94/-0.90 | 0.82/0.81 |
| Social impact^#^ | 0.57/0.56 | 0.25/0.25 | 0.81/0.81 | 0/0 | 4/4 | 1.81/1.96 | 2.92/3.61 | 0.83/0.81 |
| Psychological impact^†^ | 0.87/1.02 | 0.67/0.60 | 0.85/1.00 | 0/0 | 4/4 | 1.32/1.14 | 1.21/0.48 | 0.85/0.81 |
| Aesthetic concern | 0.70/0.81 | 0.33/0.33 | 0.95/1.01 | 0/0 | 4/4 | 1.55/1.33 | 1.75/1.09 | 0.89/0.91 |

#For Finnish sample, items 9, 13, 14 and 15 were not considered for the calculation of the score. †For Brazilian sample, item 6 was not considered for the calculation of the score.
