# Supplementary material for: Aesthetic dental treatment, orofacial appearance, and life satisfaction of Finnish and Brazilian adults
Source: PLoS One. 2023 Jun 29;18(6):e0287235. doi: 10.1371/journal.pone.0287235 (PMC10310051; doi:10.1371/journal.pone.0287235)
Supplement: S3 Table — (DOCX) [file pone.0287235.s003.docx]

**S3 Table. Principal component analysis (PCA) and parallel analysis results for Finnish and Brazilian sample.**

|  | Finnish Sample (n=3,614) | | | | Brazilian Sample (n=3,979) | | | |
| --- | --- | --- | --- | --- | --- | --- | --- | --- |
|  | PCA | | Parallel Analysis | | PCA | | Parallel Analysis | |
| Dimensions | Real-data eigenvalues | Proportion of variance | Mean of random eigenvalues | 95^th^ percentile of random eigenvalues | Real-data eigenvalues | Proportion of variance | Mean of random eigenvalues | 95^th^ percentile of random eigenvalues |
| 1 | 3.71* | 0.74 | 1.05 | 1.07 | 3.72* | 0.74 | 1.04 | 1.06 |
| 2 | 0.62 | 0.12 | 1.02 | 1.04 | 0.58 | 0.12 | 1.02 | 1.03 |
| 3 | 0.25 | 0.05 | 1.00 | 1.01 | 0.29 | 0.06 | 1.00 | 1.01 |
| 4 | 0.23 | 0.05 | 0.98 | 0.99 | 0.26 | 0.05 | 0.98 | 0.98 |
| 5 | 0.18 | 0.04 | 0.95 | 0.97 | 0.15 | 0.03 | 0.96 | 0.97 |

*Suggesting component to be retained by Parallel Analysis (real-data eigenvalues >mean of random eigenvalues)
